# *Mycoplasma bovis* Infections in Free-Ranging Pronghorn, Wyoming, USA

**DOI:** 10.3201/eid2612.191375

**Published:** 2020-12

**Authors:** Jennifer L. Malmberg, Donal O’Toole, Terry Creekmore, Erika Peckham, Hally Killion, Madison Vance, Rebecca Ashley, Marguerite Johnson, Christopher Anderson, Marce Vasquez, Douglas Sandidge, Jim Mildenberger, Noah Hull, Dan Bradway, Todd Cornish, Karen B. Register, Kerry S. Sondgeroth

**Affiliations:** Wyoming State Veterinary Laboratory, Laramie, Wyoming, USA (J.L. Malmberg, D. O’Toole, H. Killion, M. Vance, R. Ashley, M. Vasquez, T. Cornish, K.S. Sondgeroth);; University of Wyoming Department of Veterinary Sciences, Laramie (J.L. Malmberg, D. O’Toole, M. Johnson, C. Anderson, D. Sandidge, T. Cornish, K.S. Sondgeroth);; Wyoming Game and Fish Department, Laramie (T. Creekmore); Wyoming Game and Fish Department, Gillette, Wyoming, USA (E. Peckham);; Wyoming Public Health Laboratories, Cheyenne, Wyoming, USA (J. Mildenberger, N. Hull); Washington Animal Disease Diagnostic Laboratory, Pullman, Washington, USA (D. Bradway);; US Department of Agriculture National Animal Disease Center, Ames, Iowa, USA (K.B. Register)

**Keywords:** Mycoplasma bovis, mycoplasma pneumonia, pronghorn, antelope pneumonia, viruses, respiratory infections, zoonoses, Wyoming

## Abstract

*Mycoplasma bovis* is 1 of several bacterial pathogens associated with pneumonia in cattle. Its role in pneumonia of free-ranging ungulates has not been established. Over a 3-month period in early 2019, »60 free-ranging pronghorn with signs of respiratory disease died in northeast Wyoming, USA. A consistent finding in submitted carcasses was severe fibrinosuppurative pleuropneumonia and detection of *M. bovis* by PCR and immunohistochemical analysis. Multilocus sequence typing of isolates from 4 animals revealed that all have a deletion in 1 of the target genes, *adh-1*. A retrospective survey by PCR and immunohistochemical analysis of paraffin-embedded lung from 20 pronghorn that died with and without pneumonia during 2007–2018 yielded negative results. These findings indicate that a distinct strain of *M. bovis* was associated with fatal pneumonia in this group of pronghorn.

The bacterium *Mycoplasma bovis* is an economically important pathogen of cattle that contributes to the multifactorial bovine respiratory disease complex. In addition to causing respiratory disease, this bacterium can cause polyarthritis, mastitis, otitis media, and a chronic pneumonia–polyarthritis syndrome, impacting beef and dairy cattle worldwide (*1*). Despite increased recognition of its role in economic loss in the cattle industry, *M. bovis* remains a clinical challenge because of a common carrier state in clinically healthy animals, variable disease expression, intermittent shedding, and the lack of rapid accurate diagnostic assays (*1*,*2*). 

Clinical disease is not considered necessary to maintain *M. bovis* in populations, and *M. bovis* is commonly detected in asymptomatic adult feedlot cattle (*2*). Although the upper respiratory tract mucosa is a primary site for *M. bovis* colonization, presence of the bacterium in the lung is variable in occurrence and clinical manifestation. In 1 study, *M. bovis* was detected in 46% of cattle with normal lungs, 82% of cattle with acute fibrinous pneumonia, and 98% of cattle with chronic pneumonia (*3*). Manifestation of *M. bovis*–associated respiratory disease is particularly common in the wake of stress (e.g., from transportation, comingling, feedlot entry, and harsh temperatures or conditions).

In the early 2000s, *M. bovis* caused several high-mortality (case-fatality rate 45%) epizootics in bison (*Bison bison*) in North America (*4*). These events raised concern about emergent virulent strains, and research began to characterize isolates from different host species (*5*). An important difference between outbreaks of mycoplasmosis in bison and cattle is that, in the former, few or no co-infecting bacterial or viral pathogens are consistently detected (*4*,*6*–*8*). Although *M. bovis* virulence factors are poorly defined, evasion of immune response is implicated in maintaining chronic infection (*9*). One study found that that up to 79% of bison herds in western Canada have >1 *M. bovis*–seropositive animal and that 8 of 11 herds with no history of *M. bovis* disease had seropositive animals (*10*). These findings suggest that host response to *M. bovis* varies; some exposed bison become subclinical carriers and might also indicate a strain variation in *M. bovis* that influences the severity of disease.

Despite its recent recognition in bison, documented cases of *M. bovis* in free-ranging ruminants are rare. *M. bovis* was reported in farmed white-tailed deer (*Odocoileus virginianus*) (*11*) and observed in free-ranging mule deer (*Odocoileus hemionus*) (P. Wolff, Wildlife Disease Association, pers. comm., August 2019). Pronghorn (*Antilocapra americana*) are the only extant member of the family *Antilocapridae* and are native to expansive ranges in the western United States, southern Canada, and northern Mexico (*12*). Approximately 0.5–1.0 million pronghorn exist in North America (*12*). Herds are commonly sympatric with range cattle and ranched bison. In this article, we document *M. bovis* as the cause of a high-mortality outbreak of respiratory disease in a new free-ranging host, using a widely employed multilocus sequence typing (MLST) scheme to characterize associated lesions and the allelic profile of *M. bovis*.

## Materials and Methods

### Diagnostic Workup

**Figure 1:**
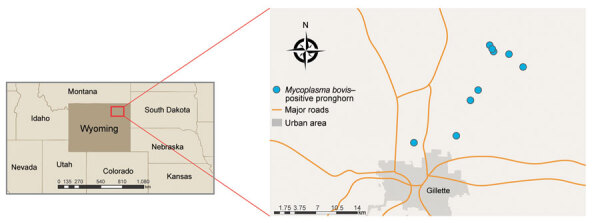
Locations of deaths in free-ranging pronghorn attributable to *Mycoplasma bovis* infection, Wyoming, USA, February–April 2019. Infections were geographically confined to northeast of state (demarcated in inset map).

Carcasses, tissue samples, or both were obtained from the site of the outbreak comprising a »20-km^2^ area northeast of Gillette, Wyoming, USA (Figure 1). The samples were submitted on behalf of the Wyoming Game and Fish Department (WGFD) for a diagnostic workup. For 2 of the 9 cases, an entire carcass was submitted and a detailed postmortem examination was performed by a board-certified pathologist, including histopathologic examination of tissues. For 7 of the 9 cases, limited tissue sampling was performed during field autopsy by WGFD. In all 9 cases, fresh or fresh frozen lung tissue was received, and *M. bovis* was detected by PCR. Bacteriology (aerobic and anaerobic culture) was performed on fresh or fresh frozen lung from 5 of 9 cases; 4 cases had advanced tissue autolysis and were not cultured. No consistent bacterial co-infections were detected across multiple cases (Table 1). Because *Mannheimia* spp. and *Histophilus* spp. were detected by culture in 1 case, we performed PCR for both agents on all 9 cases, yielding negative results. Molecular virology was performed on fresh or fresh frozen lung from all 9 cases; PCR assays included bovine herpesvirus 1, parainfluenza virus 3, bovine viral diarrhea virus, bovine respiratory syncytial virus, epizootic hemorrhagic disease virus, blue tongue virus, and a cervid adenovirus originally identified in mule deer and occasionally detected in pronghorn (*13*). No viruses were detected in any case. PCR assays were performed according to validated diagnostic protocols at the Wyoming State Veterinary Laboratory (WSVL). Histopathologic and immunohistochemical (IHC) analysis were performed on a total of 5 cases. In all cases, *M. bovis* was detected by IHC analysis. IHC analysis for *Histophilus somni* was also performed on these 5 cases; *H. somni* was not detected.

**Table 1:** Summary of pronghorn cases associated with pneumonia outbreak, Wyoming, USA, 2019*

Case no.	Age category and sex	Sample type	Histopathologic results	*Mycoplasma bovis* IHC result	*M. bovis* PCR test result	Aerobic culture	*M. bovis* culture
1	Adult male	Lung, kidney, liver, spleen, bone marrow	Exudative pneumonia	Detected	Detected	*Trueperella pyogenes*	Positive
2	Adult female	Lung	Pleuropneumonia with caseous abscesses	Detected	Detected	No growth	Positive
3	Adult female	Whole carcass	Bronchointerstitial pneumonia, fibrinonecrotic and suppurative with fibrinous pleuritis	Detected	Detected	Mixed bacteria	Positive
4	Adult female	Lung, kidney, liver, spleen	Pleuropneumonia with caseonecrotic abscesses	Detected	Detected	Mixed bacteria	Positive
9	Yearling male	Whole carcass	Bronchointerstitial pneumonia, fibriononecrotic and suppurative with fibrinous pleuritis, caseonecrotic abscesses, lymphocytic cuffing	Detected	Detected	*Mannheimia spp.,* *Histophilus spp.*	Positive
5	Adult female	Lung	NE	NA	Detected	NA	Positive
6	Adult female	Lung, kidney, liver, spleen	NE	NA	Detected	NA	Positive
7	Adult female	Lung	NE	NA	Detected	NA	Positive
8	Adult female	Lung	NE	NA	Detected	NA	Positive

*IHC, immunohistochemical; NA, not applicable; NE, not examined.

### Histopathology

Tissues collected at autopsy for histopathologic analysis (Appendix) were fixed in 10% buffered formalin and processed conventionally before embedding in paraffin wax. Sections cut at 5 µm were stained with hematoxylin and eosin. IHC analysis of *M. bovis* and *H. somni* was performed on lung tissue derived from the same blocks as used in the hematoxylin and eosin assays (Appendix).

### Mycoplasma Culture

Approximately 20 mg of lung tissue was placed in a mycoplasma enrichment broth (Hardy Diagnostics’ Mycoplasma Broth; Hardy Diagnostics, https://hardydiagnostics.com) and incubated with a loose lid at 37°C in 10% CO_2_ for 72 h. Subsequently, 100 μL of broth was inoculated onto a commercial *Mycoplasma* spp. medium (Hardy Diagnostics’ Mycoplasma Agar with Cefoperazone) and spread evenly over the entire plate with a sterile swab. Plates were incubated at 37°C in 10% CO_2_ for 72–240 h, depending on appearance of colony growth. Colonies from each isolate were analyzed by matrix-assisted laser desorption/ionization-time of flight mass spectrometry (Bruker’s Biotyper, https://www.bruker.com) according to the manufacturer’s instructions for identification. Additional colonies were used for whole-genome sequencing.

### Sequencing

#### 16S 

After DNA extraction from fresh lung tissue, a portion of the 16S ribosomal RNA gene was amplified by PCR using universal *Mycoplasma* primers (*13*) at the Washington Animal Disease Diagnostic Laboratory. PCR amplicons were directly sequenced, and a GenBank BLAST search was performed (https://blast.ncbi.nlm.nih.gov) on consensus sequence from 2 forward and 2 reverse high-quality reads. This initial confirmation was performed on the first sample only, and isolates from subsequent samples were confirmed by whole-genome sequencing.

#### Whole-Genome Sequencing

Short-read sequencing technology was used on extractions of pure *M. bovis* isolates. Postsequencing statistics were evaluated by using FastQC (*14*) (Appendix).

### Polymerase chain reaction

#### Diagnostic *M. bovis* PCR

DNA was extracted from fresh lung tissue and PCR was performed targeting the *M. bovis* 16S ribosomal RNA gene (Appendix). Confirmation of diagnosis from case 1 (Table 1) by PCR targeting of the *uvrC* gene was performed at the Washington Animal Disease Diagnostic Laboratory (*15*).

#### Survey of Formalin-Fixed Paraffin-Embedded Archival Lung Tissue

DNA was extracted from formalin-fixed, paraffin embedded lung tissue curls cut at a thickness of 20 μm. In brief, 1–2 curls per sample were dewaxed by using xylene and ethanol according to the DNeasy Blood and Tissue kit’s recommended protocol (QIAGEN, https://www.qiagen.com). The tissue extraction proceeded overnight at 56°C, according to manufacturer instructions. Cases selected for PCR were based on the availability of lung tissue from pronghorn in archived wax blocks, which are retained for 15 years because of limited storage space. A total of 20 cases (13 in pronghorn with previously diagnosed pneumonia) were identified; all cases originated from Wyoming (Table 2). After DNA extraction, PCR was performed as described previously for the diagnostic *M. bovis* PCR assay.

**Table 2:** Pronghorn with and without pneumonia, Wyoming, USA, 2007–2019

Case no.	Year	Pneumonia	Other diagnosis	Geographic area
1	2007	Yes	None	Southeast
2	2014	Yes	None	Southeast
3	2014	Yes	*Trueperella pyogenes*	South central
4	2015	Yes	*Corynebacterium spp.*	South central
5	2015	Yes	*T. pyogenes*	Central
6	2016	Yes	*T. pyogenes*	West central
7	2016	Yes	None	Northeast
8	2016	Yes	*Protostrongylus spp.* lungworms, *Dermacentor spp.* ticks, *Haemonchus contortus* abomasal worms	Southeast
9	2016	Yes	*Dermatophilus congolensis*	Southeast
10	2017	Yes	*T. pyogenes*	Central
11	2017	Yes	*Dictyocaulus spp.*	Southeast
12	2018	Yes	Epizootic hemorrhagic disease virus	East central
13	2018	Yes	*T. pyogenes*	Central
14	2018	No	Blackleg from *Clostridium chauvoei*	Southeast
15	2018	No	None	West central
16	2018	No	Bluetongue virus	Southeast
17	2018	No	Peritonitis	Southeast
18	2018	No	None	Southeast
19	2019	No	Foot defect	West central
20	2019	No	Trauma from hail	Southeast

#### Diagnostic *M. ovipneumoniae* PCR

A 2 × 2 cm section of fresh lung tissue was placed into 2 mL modified tryptic soy broth and homogenized for 120 seconds. The homogenous solution was transferred to a snap cap tube and incubated at 37°C with 10% CO_2_ for 48 h. After centrifugation of 1 mL, the pellet was resuspended and used in a PCR reaction as previously described (*16*).

#### *adh-1* PCR

DNA was extracted from the *Mycoplasma* broth of each sample stored at −80°C by using the QIAGEN DNeasy Blood and Tissue Kit fluid protocol. In brief, 200 μL was extracted following manufacturer instructions. Forward and reverse primers targeting the *adh-1* gene (0.5 μmol/L of each) were used in a 50 μL reaction containing 22.5 μL GoTaq green master mix (Promega, https://www.promega.com), 1.5 μL of 50 nM MgCl_2_, and nuclease-free water (*5*).

### MLST Analysis

Paired fastq reads of »250 bp were analyzed as follows: trimming of indexes, primers, low quality (phred <20), and short reads (<50 bp) using Cutadapt (*17*); mapping of trimmed reads to the genome of *M. bovis* international reference strain PG45 (GenBank accession no. NC_014760) using Bowtie2 (*18*); conversion of .sam files to .bam files using Samtools (*19*); and viewing of sorted .bam files in Geneious Prime 2019.1.3 (https://www.geneious.com). Consensus sequences were generated from mapped reads by using the highest quality parameter in Geneious Prime as a threshold. “N” was assigned to sites with coverage <3 to represent missing data. Consensus sequences were trimmed to loci employed in the MLST scheme described by Register et al. (*5*) and concatenated in frame. Concatenated sequences were compared for 4 *M. bovis* isolates recovered from the lung samples of 4 different pronghorn across the following MLST genes: alcohol dehydrogenase (*adh-1*), glutamate tRNA ligase (*gltX*), glycerol-3-phosphate dehydrogenase (*gpsA*), DNA gyrase subunit B (*gyrB*), phosphate acetyltransferase-2 (*pta-2*), thymidine kinase (*tdk*), and transketolase (*tkt*) (*5*). Isolates derived in our study were compared with those from the University of Oxford *Mycoplasma bovis* MLST website (https://pubmlst.org/mbovis) (*20*). An aligned fasta file was obtained for all publicly available isolates missing the *adh-1* gene (i.e., nontypeable isolates). The representative sequence from the 4 identical pronghorn isolates was aligned to the fasta file comprising all nontypeable isolates by using Muscle (*21*), and model selection was performed in MEGA X (*22*). Sequence alignments were subjected to maximum-likelihood phylogenetic analyses under the Hasegawa–Kishono–Yano substitution model using PhyML (*23*) with 10,000 bootstrap replicates for support.

## Results

### Disease Outbreak

At least 60 pronghorn died during February–April 2019 within a total area of »13 km^2^. WGFD received initial reports of »30 carcasses in early February 2019. An additional 20 pronghorn deaths were identified within 8 km of the site of initial reports within 1 month, and the affected area expanded as wintering herds began to disperse with warmer weather. In March, a herd of »10 pronghorn moved to the same area and began dying within 2 weeks. Landowners reported that affected pronghorn appeared lethargic. Such animals were typically dead within 24 hours. Because of logistics of finding fresh carcasses in remote areas on private land during 2 major winter storms, only a fraction of the dead pronghorn could be sampled. Two carcasses were obtained for autopsy, and tissues were obtained from an additional 3 cases for histopathologic examination and PCR. Samples were collected from 4 additional animals for PCR only. The extent of the die-off could not be estimated until improved weather conditions allowed WGFD biologists to conduct ground and aerial surveys. Bison, cattle, or other free-ranging ungulates (i.e., deer, elk, and moose) deaths associated with pneumonia were not reported in the area during this outbreak. The closest captive bison herd was located »64 km south of the outbreak site. Although pronghorn deaths occur in winter because of starvation, predation, and vehicular collision, 60 deaths in a small area is unusual.

### Diagnosis and Characterization of Lesions

**Figure 2:**
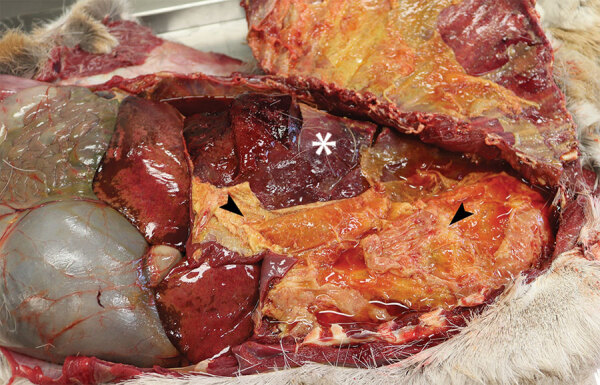
Free-ranging pronghorn infected with *Mycoplasma bovis* with severe fibrinous pleuropneumonia, Wyoming, USA, February–April 2019. Open thoracic cavity with ribs reflected reveals abundant fibrin on the visceral pleura (arrowhead) and consolidated lungs (asterisk).

**Figure 3:**
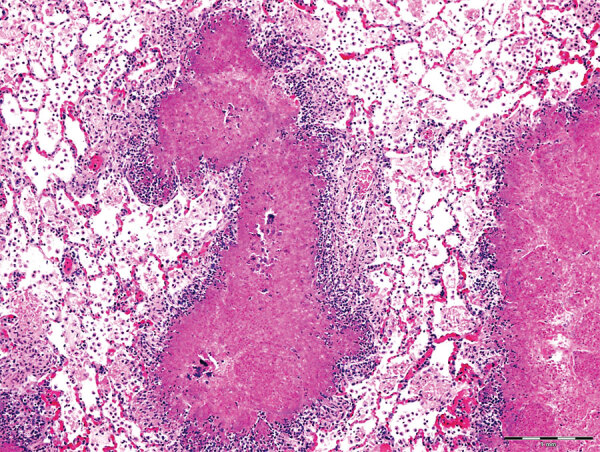
Histologic lung lesions in free-ranging pronghorn, characterized by caseonecrotic foci centered on residual bronchioles, Wyoming, USA, February–April 2019. Alveolar fibrin exudation and suppurative to mixed inflammation throughout. Scale bar indicates 1 mm.

**Figure 4:**
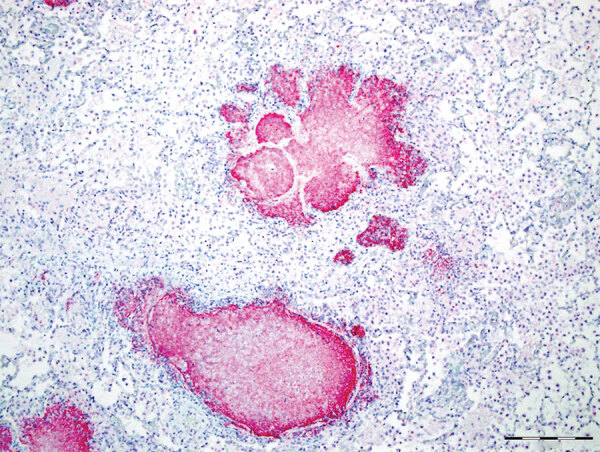
Caseonecrotic lung lesions in free-ranging pronghorn found to be strongly immunopositive for *Mycoplasma bovis* antigen by immunohistochemical analysis, Wyoming, USA, February–April 2019. Positive staining indicated by fast red coloring has strong intensity and specificity for lesions centered on bronchioles. Scale bar indicates 1 mm.

Gross lesions were characterized by severe, regionally extensive to diffuse, bilateral fibrinous pleuropneumonia affecting an estimated 50%–100% of lung parenchyma (Figure 2). Histopathologic examination revealed fibrinosuppurative pneumonia with caseonecrotic foci centered on bronchi and bronchioles. Caseonecrotic foci were characterized by central granular eosinophilic material and necrotic leukocytes surrounded by degenerate and intact neutrophils. Some foci were partly mineralized (Figure 3). Lesions were interpreted as acute to subacute, because features of chronic infection, such as extensive fibrosis, were absent (*24*). In all 5 cases for which histopathologic examination was performed, pulmonary lesions were strongly immunoreactive for *M. bovis* antigen, with characteristically strong staining at the margins of necrotic foci as described in affected cattle and bison (*3*) (Figure 4). The pronghorn in case 9 (Table 1) had pulmonary abscesses up to 1 cm diameter characterized by coagulative necrosis surrounded by a thin band of fibrosis. Features characteristic of mannheimiosis, histophilosis, or both, such as neutrophils with oat cell morphology, were absent. The pronghorn in case 9 also had fibrinosuppurative synovitis and conjunctivitis. *M. bovis* antigen was detected in the conjunctiva by IHC analysis. In addition, acute centrilobular hepatic necrosis was identified in case 9. We attributed this finding to hypoxia secondary to severe pneumonia.

Lung samples from the 9 pronghorn cases were positive for *M. bovis* by culture and PCR (Table 1). 16S sequencing revealed that the isolate from case 1 most closely matched that of *M. bovis* (100% sequence identity [553/553 bp]; GenBank no. KX462388). The next closest match was 99% identity (549/553 bp) to *M. agalactiae* (GenBank no. AF332750). *M. ovipneumoniae* PCR was performed on samples of lung; all results were negative (data not shown).

Aside from the consistent detection of *M. bovis*, aerobic culture results were inconsistent. The pronghorn in case 1 contained *Trueperella pyogenes*, the pronghorn in case 9 contained both *Mannheimia* spp. and *Histophilus* spp., and the pronghorn in cases 3 and 4 had a mixture of bacterial species not typically associated with pneumonia interpreted as incidental (Table 1). The pronghorn in cases 2 and 9 had a mild lungworm infection, including nematode larvae and eggs histologically consistent with *Dictyocaulus* spp. parasitic infection.

### Retrospective Study

We performed a retrospective survey for *M. bovis* on 20 archived WSVL cases of pronghorn deaths with and without pneumonia (Table 2); embedded lesioned lung from the animals in 2 of the 9 positive cases among the 2019 pronghorn deaths were used as positive controls. All archived pronghorn lung tissues were negative for *M. bovis* by PCR and IHC analysis, and none had lesions suggestive of mycoplasmosis.

### MLST and Phylogenetics

Genome sequencing of the 4 *M. bovis* isolates recovered from the pronghorn carcasses was performed at the Wyoming Public Health Laboratory. All isolates from pronghorn had 100% sequence identity at loci used for MLST (*5*). All assemblies contained an apparent deletion of 1 of the 7 MLST target genes, *adh-1* (*5*). Sequences for the 6 remaining loci are available through GenBank (accession nos. MT782331–6). To confirm the *adh-1* deletion, DNA from *M. bovis* pronghorn isolates was amplified by PCR using the *adh-1* primers specified for MLST as described previously (*5*). DNA extracted from a cattle isolate of *M. bovis* was strongly positive, whereas DNA from the pronghorn isolates produced no visible band upon gel electrophoresis.

**Figure 5:**
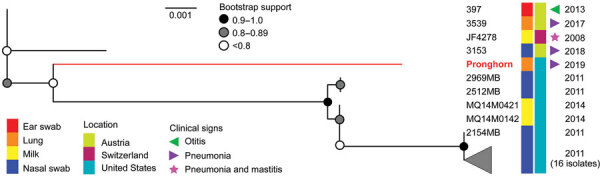
Phylogeny of *Mycoplasma bovis* isolates from free-ranging pronghorn (red branch), Wyoming, USA, February–April 2019. Pronghorn were found to be divergent from all bovine isolates with a deletion of *adh-1* gene but are most similar to those recovered from cattle in the United States. This unrooted maximum-likelihood tree (10,000 bootstrap replicates) comprises all available nontypeable isolates and is based on 6 of 7 sequence typing loci. The health status of cattle sampled during 2011–2014 is unknown, and the absence of reported clinical signs does not necessarily equate to absence of disease. Scale bar indicates substitutions per site.

Deletion of *adh-1* has been identified in *M. bovis* isolates derived from cattle (*25*; https://pubmlst.org/mbovis; K.B. Register, unpub. data). Phylogenetic analysis of these MLST-nontypeable isolates based on DNA sequences of the other 6 MLST targets revealed that the pronghorn isolates we evaluated are divergent from all others typed to date but are most similar to a group of isolates obtained from cattle in the United States since 2011 (Figure 5).

## Discussion

*M. bovis* is uncommon in free-ranging ungulates. Accounts are limited to cases in farmed white-tailed deer (*11*), and free-ranging mule deer (*26*; P. Wolff, Wildlife Disease Association, pers. comm., August 2019). Lesions in lung were compatible with the lesions attributable to *M. bovis* in cattle and bison (*2*,*6*). The distribution of *M. bovis* antigen in IHC preparations of caseonecrotic foci is typical of fatal mycoplasmosis in cattle (*2*,*3*).

To determine whether *M. bovis* had been previously overlooked in Wyoming pronghorn, we queried the WSVL diagnostic database. We identified 20 cases from different geographic regions of Wyoming that occurred during 2007–2019. This group included 13 cases in pronghorn with previously diagnosed pneumonia and 7 without (Table 2). Although the *M. bovis* PCR assay used at WSVL has not been validated for formalin-fixed, wax-embedded tissue, positive dewaxed lung samples from pronghorn in the 2019 cases were used as a control. On the basis of these 20 samples, no *M. bovis* infections in pronghorn before 2019 was evident.

Draft genome sequences were obtained for isolates of *M. bovis* from 4 pronghorn in the 2019 group. Compared with MLST data available on >700 isolates, only 9 complete genome assemblies from other host species, such as goat, bison, or cattle, were available. Thus, we determined sequence type by extracting regions of 7 genes used in MLST typing (*5*). The 4 isolates were identical across these loci and contained a deletion encompassing the *adh-1* gene. This deletion was confirmed by using *adh-1* specific primers as described previously (*5*). These isolates were compared with others with a deletion at the *adh-1* locus (i.e., nontypeable isolates). Although the *adh-1* gene deletion has been identified in *M. bovis* from bison and mule deer (K.B. Register, unpub. data), only sequences from bovine isolates are currently available in the *Mycoplasma bovis* pubMLST database (https://pubmlst.org/mbovis). The isolates from pronghorn are divergent from other published isolates but are most similar to those from US cattle compared with bovine isolates from Austria or Switzerland (Figure 5).

Although the *adh-1* deletion has not yet been thoroughly characterized, the earliest identification of this variant is from 2008. The deletion might be relatively recent and might be associated with expansion of *M. bovi*s host range or emergence in new species. Additional research is needed to investigate the possible association between the *adh-1* gene deletion and the recent appearance of *M. bovis* in pronghorn. It will be of interest to investigate the entire genome for other whole-gene deletions or insertions and to correlate whether genomic changes are associated with certain hosts, levels of virulence, or both.

Surveillance of pronghorn samples submitted to WSVL has not identified *M. bovis* in other areas of the state at this time. No additional cases have been diagnosed in northeast Wyoming since April 2019. As part of a surveillance effort, we have recently performed *M. bovis* PCR on lung tissue DNA of any ungulate submitted to WSVL. We have found no evidence of chronically infected pronghorn or other wildlife reservoirs of this bacterium. The host species of origin in this outbreak is unknown. Given the frequency of *M. bovis* in asymptomatic cattle and bison and its rarity of detection in free-ranging ungulates, transmission to pronghorn from a livestock reservoir seems likely.

Our findings strongly implicate *M. bovis* as a primary pathogen in pronghorn, resulting in fatal pneumonia in absence of other respiratory pathogens with changes comparable to those in bison with fatal mycoplasmosis. *M. bovis* as a primary pathogen in bison is in contrast to *M. bovis* in adult cattle, where the bacterium tends to occur most commonly as 1 component of chronic, polymicrobial respiratory disease (*24*,*27*). The pronghorn *M. bovis* infection is more analogous to mycoplasmosis in bison, where it is known to be a primary cause of pneumonia, arthritis, pharyngitis, and reproductive disorders (*6*–*8*).

Our findings document *M. bovis* infection in pronghorn and highlight the possible health implications for other wildlife populations and the potential risk for transmission at the wildlife–livestock interface. Furthermore, we document *M. bovis* genetic variation in association with virulent disease in pronghorn, supporting hypotheses that *M. bovis* might be expanding in host range and in disease expression. We therefore recommend that *M. bovis* be considered as a differential diagnosis for pneumonia in wildlife, particularly in outbreak scenarios. Traditionally, pronghorn are not considered a major source of disease threats to either cattle or bison and are therefore widely tolerated on commercial operations. Additional studies are needed to establish transmission potential and direction, which will elucidate the importance of *M. bovis* at the wildlife–livestock interface.

Appendix Additional information about *Mycoplasma bovis* infections in free-ranging pronghorn, Wyoming, USA.
