# Supplementary material for: Mycoplasma bovis Infections in Free-Ranging Pronghorn, Wyoming, USA
Source: Emerg Infect Dis. 2020 Dec;26(12):2807–14. doi: 10.3201/eid2612.191375 (PMC7706946; doi:10.3201/eid2612.191375)
Supplement: Appendix — Additional information about Mycoplasma bovis infections in free-ranging pronghorn, Wyoming, USA. [file 19-1375-Techapp-s1.pdf]

Article DOI: https://doi.org/10.3201/eid2612.191375

Mycoplasma bovis Infections in Free-Ranging Pronghorn, Wyoming, USA

Appendix

Methods

Histopathology

Tissues for Histopathology included lung only (n = 1 case); lung, liver, kidney and spleen (n = 1 case); lung, liver, kidney, spleen and bone marrow (n = 1 case); lung, liver, kidney, spleen, heart, brain, lymph node, abomasum, omasum, reticulum, rumen, small intestine, colon, trachea, esophagus, thyroid gland, salivary gland, adrenal gland, tongue, skeletal muscle placenta and bone marrow (n = 1 case); and lung, liver, kidney, spleen, heart, brain, lymph node, abomasum, omasum, reticulum, rumen, small intestine, colon, adrenal gland, skeletal muscle, bone marrow, synovium and third eyelid (n = 1 case).

Immunohistochemistry

For *M. bovis* immunohistochemistry (IHC), a polyclonal rabbit-origin primary antibody (provided by Karen Sverlow, UC Davis), and a commercial immunostainer (intelliPATH FLX; Biocare, Pacheco, CA, USA) were utilized. For antigen retrieval, sections were pre-treated with a pH 6.0 citrated antigen-retrieval solution (Diva Decloaker 10X, Biocare) and a digital electric pressure cooker (Decloaking Chamber, Biocare) for 20 minutes at 121°C. Nonspecific background staining was quenched (Background Punisher, Biocare). The primary antibody was used at a dilution of 1:12,000 for 1 hour, followed by a biotin-free alkaline phosphatase anti-rabbit kit (Rabbit-on-Canine AP-Polymer, Biocare) and a Fast Red chromogen (IP Warp Red, Biocare). Sections were counterstained with hematoxylin. Specificity of staining was confirmed using validated positive tissue (bovine lung with *M. bovis*, detected by PCR of fresh tissue), and an omit control where the anti-*M. bovis* antibody was replaced by a universal negative control serum (Universal Negative Control Serum, Biocare). For *H. somni* IHC, the polyclonal rabbit-

origin primary antibody was provided by Lynette Corbeil, and used at a dilution of 1:1000 for 1 hour, otherwise following the same steps for *M. bovis* as described above.

Whole-Genome Sequencing

Pure sub-cultures of *Mycoplasma bovis* isolates were sequenced. Subcultures were obtained from broth cultures onto solid media, and DNA extractions were performed using the DNeasy Blood & Tissue Kit (Qiagen, Germantown, MD, USA) on an automated nucleic acid extraction platform (QIAcube, Qiagen, Germantown, MD, USA). DNA extract was assessed for purity (260/280 nm) (NanoDrop 2000, Thermo Scientific, Grand Island, NY, USA) and concentration (Qubit 3.0 fluorometer, Thermo Scientific). A DNA library was prepared using the Nextera DNA Flex library Prep Kit (Illumina, San Diego, CA, USA). The library quantity was again measured using the Qubit 3.0 fluorometer (Thermo Scientific). Length of the DNA library fragments was assessed using the TapeStation 4200 (Agilent, Santa Clara, CA, USA), and after normalization, DNA libraries were sequenced on a MiSeq using V2 2 × 250bp cycle chemistry with ≈8.0 Gb of output (Illumina, San Diego, CA, USA).

PCR from Fresh Lung Tissue

DNA from fresh tissue was extracted using the DNeasy blood and tissue kit (Qiagen, Germantown, MD, USA). Four µL of DNA extract was used in each *Mycoplasma bovis* PCR reaction targeting the 16S ribosomal RNA gene using MBVF (5′-TGA TAG CAA TAT CAT AGC GGC-3′), MBVR (5′-GTA GCA TCA TTT CCT ATG CTA C-3′) in a 50 µL reaction containing 25uL GoTaq green Master Mix (Promega, Grand Island, NY, USA), nuclease free water, 1 µL of 50 mM MgCl, and 400nm of each primer. Cycling conditions of 94°C for 3 min, 40 cycles of 94°C for 30 s, 55°C for 30 s, 72°C for 1 min, and 72°C for 5 min (*1*). An amplicon size of 415bp was evaluated on 1.5% agarose gel, and if present the case was considered positive.

Reference

1. Naikare H, Bruno D, Mahapatra D, Reinisch A, Raleigh R, Sprowls R. Development and evaluation of a novel Taqman real-time PCR assay for rapid detection of *Mycoplasma bovis*: comparison of assay performance with a conventional PCR assay and another Taqman real-time PCR assay. Vet Sci. 2015;2:32–42. PubMed https://doi.org/10.3390/vetsci2010032
